# Profiling of the Transcriptomic Responses of *Clonostachys rosea* Upon Treatment With *Fusarium graminearum* Secretome

**DOI:** 10.3389/fmicb.2018.01061

**Published:** 2018-06-07

**Authors:** Zerihun A. Demissie, Simon J. Foote, Yifang Tan, Michele C. Loewen

**Affiliations:** ^1^Aquatic and Crop Resource Development, National Research Council Canada, Ottawa, ON, Canada; ^2^Human Health Therapeutics, National Research Council of Canada, Ottawa, ON, Canada; ^3^Aquatic and Crop Resource Development, National Research Council Canada, Saskatoon, SK, Canada; ^4^Department of Biomedical and Molecular Sciences, Queens University, Kingston, ON, Canada

**Keywords:** *C. rosea*, secondary metabolites, *Fusarium* head blight, biocontrol, mycoparasitism, gene clusters, RNAseq, differential gene expression

## Abstract

*Clonostachys rosea* strain ACM941 is a fungal bio-control agent patented against the causative agent of Fusarium Head Blight, *Fusarium graminearum*. Although the molecular details remain enigmatic, previous studies have suggested that *C. rosea* may secrete *F. graminearum* growth inhibitors. Further toward this, experiments described herein show that induction of *C. rosea* cultures by the addition of an aliquot of *F. graminearum(Fg)*-spent media (including macroconidia), yield *C. rosea (Cr)*-spent media that elicited higher anti-*F. graminearum* activity than either control or deoxynivalenol (DON)-induced *Cr*-spent media. To gain additional insight into the genetic and metabolic factors modulating this interaction, transcriptomic (RNAseq) profiles of *C. rosea* in response to DON and *Fg-*spent media treatment, were developed. This analysis revealed 24,112 *C. rosea* unigenes, of which 5,605 and 6,285 were differentially regulated by DON and *F-*spent media, respectively. More than half of these unigenes were up-regulated, with annotations, most notably in the *Fg*-spent media treatment data, suggesting enhancement of polyketide (PK) and non-ribosomal peptide (NRP) secondary metabolite precursor synthesis, and PK/NRP-like synthases. Four ABC transporters were also up-regulated in response to *Fg-*spent media. Further analysis showed that the PK and NRP-like synthases belong to three gene clusters that also include ABC transporters, and other genes known to tailor secondary metabolite biosynthesis. The RNAseq data was further validated using quantitative RT-qPCR. Taken together, these results show that *C. rosea* responds to the presence of *Fg*-spent media (and to a lesser extent, DON-alone) by up-regulating unique aspects of its secondary metabolism-related genetic repertoire. The identities and roles of *C. rosea* secondary metabolites produced by the targeted gene clusters are now under investigation.

## Introduction

*Fusarium* Head Blight (FHB or scab) disease, caused by several molds of the genus *Fusarium*, is the leading cause of yield loss in cereal crops throughout the world. *Fusarium graminearum, F. culmorum, F. avenaceum*, and *F. crookwellense* are the main species causing FHB in North America, with *F. graminearum* Schwabe [teleomorph, *Gibberella zeae* (Schwabe) Petch] posing the greatest economic threat due to its aggressive nature (Hue et al., 2009; Xue et al., 2014). As part of the infection process, *F. graminearum* actively produces mycotoxins, mainly the sesquiterpene lactone deoxynivalenol (DON) and its acetyl-derivatives. These mycotoxins are deposited on the grain, both out in the field and during grain storage, resulting in significant loss of market value.

Despite *F. graminearum's* economic importance, effective FHB control and DON detoxification methods are, thus far, lacking. Both the complexity of the wheat genome and the lack of a genetic resistance pool in wheat germplasm are contributing to the bottleneck in breeding FHB resistant varieties. Indeed while over 50 QTLs associated with FHB resistance have been identified so far, each contributes only limited degrees of resistance and attempts to stack them into commercial varieties have not succeeded so far (McCartney et al., 2016). As such, best agronomic practices involving the use of partially resistant varieties, crop rotation and timely fungicide application are recommended to minimize FHB economic damage. However, the efficacy of these practices are often compromised by increasing environmental conditions favorable to pathogen sporulation and thus a general increase in FHB incidence, with no suitable post-harvest contamination controls. These shortcomings, in addition to the growing concern about the impact of chemical fungicides on the environment and pathogen resistance, have led to the identification of several biological control agents (BCAs) against FHB including for example, *Bacillus, Pseudomonas* and *Trichoderma* spp. (Palazzini et al., 2007; Comby et al., 2017). BCAs offer an environmentally safe, effective and durable alternative disease control strategy, with the added benefit of inducing plant vigor. For example, da Luz (2000) reported a 50–60% FHB severity reduction and more than 700 kg/ha increase in yield by using Brazilian isolates of *Bacillus* and *Paenibacillus* species. To date, *Pseudomonas chlororaphis* and *Pythium oligandrum* are commercially available as bio-fungicides against FHB in Europe (Comby et al., 2017).

The endophytic fungus *Clonostachys rosea* (formerly known as *Gliocladium roseum*) is a soil-borne ascomycete well regarded for its mycoparasitic properties against diverse plant pathogens including *Fusarium* spp., *Alternaria* spp., *Botrytis cinera, Pythium tracheiphilum*, and nematodes (Sutton et al., 1997; Zhang et al., 2008; Lysøe et al., 2017). *C. rosea* strain ACM941 was isolated from pea plants in Manitoba, Canada in 1994 (Hue et al., 2009) and ensuing greenhouse and field experiments confirmed that it has the capacity to reduce the FHB index by 46%, DON levels by 33% and increased yield by 7%, levels comparable to Folicur treatments (Xue et al., 2014). Also notable is ACM941's impressive *F. graminearum* perithetical production-suppressing ability when applied on crop residues, which is synonymous to a crop-rotation effect. ACM941 has been patented for commercial use against FHB (United States patents 6,495,133 and 9,603,369). Another *C. rosea* strain, IK726, isolated from barley roots in Denmark, has also shown promising bio-fungicide potential against economically important plant pathogens like *Fusarium culmorum* and *Bipolaris sorokiniana* (Knudsen et al., 1995), *Alternaria* spp. (Jensen et al., 2000), *Pythium* spp. (Møller et al., 2003) and *Tilletia tritici* (Jensen et al., 2000). However, the molecular and biochemical basis of *C. rosea* antagonist mechanisms and mycotoxin tolerance remains poorly understood.

Host-defense response induction, as observed against gray mold in tomato (Mouekouba et al., 2014), and secretion of extracellular lytic enzymes such as endochitinase (Chi67-1) against *Sclerotiania sclerotiorum* (Sun et al., 2015, 2017), are some of the proposed *C. rosea* biocontrol mechanisms. Mutation of the subtilisin-like extracellular serine protease gene prC has also demonstrated this enzymes role in *C. rosea*'s virulence against nematodes (Zou et al., 2010). However, secretion of antifungal metabolites, combined with tolerance to xenobiotics, have been proposed as the principal modes of *C. rosea*'s antagonism against *Fusarium* spp. (Hue et al., 2009; Rodríguez et al., 2011; Dubey et al., 2014; Karlsson et al., 2015). In fact, the *C. rosea* genome harbors a large repertoire of putative biosynthetic gene clusters encoding a plethora of secondary metabolite synthases, including mainly polyketides (PKs) and non-ribosomal peptides (NRPs) (Karlsson et al., 2015; Sun et al., 2015). In this light, it is notable that *C. rosea* IK726 was recently shown to secrete an as-yet unidentified heat stable antifungal metabolite in liquid culture, where deletion of its pdr5-homolog attenuated the growth inhibition property of its culture filtrate and impaired its ability to antagonize *F. graminearum* (Dubey et al., 2014). Notably, pdr5's are a family of pleitropic drug resistance transporter 5 homologs, that are expected to have broad transport allocrite specificity, and in *C. rosea* was also found to be up-regulated by zearalenone, while its deletion rendered mutant cells sensitivity to this mycotoxin (Dubey et al., 2014). However, the molecular and biochemical basis of *C. rosea's* antagonistic mechanisms and the role of its extensive secondary metabolism-related genetic pool during the *C. rosea vs. F. graminearum* interaction remain enigmatic.

To date, there is only a single report highlighting the partial purification of one non-ribosomal peptide from *C. rosea* (strain BAFC3874 culture filtrate), which was toxic against *S. sclerotiorum in vitro* (Rodríguez et al., 2011). Another more recent study investigated the molecular response of *C. rosea* strain IK726 upon DON exposure in liquid medium, but identified only a limited number of potential secondary metabolism-related genes, in contrast to *C. rosea's* known genome composition (Dubey et al., 2014; Kosawang et al., 2014). This could in part be due to the inherent limitations of the EST library construction method used, but it is also likely that DON alone is not sufficient to trigger the over-expression of secondary metabolite biosynthesis-related genes under laboratory conditions. In agreement with this, other reports have shown that fungi generally activate their tightly regulated secondary metabolite production machinery only in fermentation conditions mimicking the chemical and/or microbial diversity of their natural ecology (Marmann et al., 2014; Reen et al., 2015; Okada et al., 2017). For example: the secondary metabolite biosynthetic ability of the endophyte *Silybum marianum* was activated by supplementing the fermentation medium with autoclaved leaves and plant extracts of the host plant (El-Elimat et al., 2014). As well, growth of *C. rosea* strain 67-1 along with live *S. sclertiorum* sclerotia was found to yield a more reliable *in vivo* representative RNAseq library (Sun et al., 2015).

In this study, RNAseq libraries of *C. rosea* ACM941 treated with *F. graminearum* strain GZ3639 culture filtrate containing live macroconidias, DON alone, and methanol alone (DON solvent) were developed and analyzed, to gain insight into the mechanism and biochemistry of its antifungal metabolite production machinery in liquid culture. Results reported herein show that although the overarching transcriptomic level responses of *C. rosea* to DON and *F. graminearum* strain GZ3639 culture filtrate containing live macroconidias are similar, secondary metabolism-related transcripts are exclusively up-regulated by *F. graminearum* strain GZ3639 culture filtrate containing live macroconidias. In particular, the up-regulation of three gene clusters built around polyketide and non-ribosomal peptide synthases were identified only in the spent medium induced library. Furthermore the observed enhanced antifungal activity of spent medium-treated *C. rosea* culture filtrate implies that the products of these clusters could serve as biological antifungals or could be involved in the protection of the fungi. Overall, understanding the genetic and molecular basis of the antifungal properties of *C. rosea*-specific secondary metabolites, will lead to the development of more potent biocontrol agents and/or more potent and specific lead antifungal compounds, for use against *Fusarium* in the future.

## Methods

### Fungal strains and general culture propagation and macroconidia production

*Clonostachys rosea* (Link:Fr.) Schroers, Samuels, Serfert, and Gams (syn. *Gliocladium roseum* Bainier) strain ACM941 (American Type Culture Collection ATCC 74447) was obtained from Dr. Allen Xue's laboratory (Ottawa Research and Development Centre ORDC) with permission from the patent holder, Adjuvant Plus Inc. (Kingsville, Canada), grown at 25°C for 10–12 days and maintained on potato dextrose agar (PDA) plates at 4°C. *Fusarium graminearum* strain GZ3639 (Proctor et al., 1995) was kindly provided by Dr. Susan McCormick (USDA, Peoria, IL), grown at 25°C for 4–5 days and maintained on PDA plate at 4°C. DON was purchased from Sigma-Aldrich (Oakville, ON) and dissolved in methanol at 5 mg/ml. For long term storage, agar plugs (5–6) of the growing edge of the fungus were collected in Eppendorf tubes and overlaid with 20% glycerol, flash frozen in liquid nitrogen and stored at −80°C.

*F. graminearum* macroconidia were produced by inoculating *F. graminearum* mycelia into carboxymethylcellulase (CMC) media (NH4NO3: 1 g/L; KH2PO4: 1 g/L; MgSO4. 7H_2_O: 0.5 g/L; Yeast Extract: 1.0 g/L and CMC: 15 g/L) and growing for 10 days at 25°C. Following mycelial removal by filtering the media through 4–5 layers of cheesecloth, macroconidia were pelleted by centrifugation at 4,000 × g for 20 min at 4°C and washed twice using sterile-water. Macroconidia were re-suspended in 0.5–1 mL sterile-water and the concentration determined by counting using a hemocytometer. The final concentration of macroconidia in this stock was adjusted to 10^7^/mL using sterile-water.

### Generation of secretome extracts and growth inhibition assays

The *Fg*-spent media extract (secretome) was prepared as follows. Four agar plugs from the growing edge of *F. graminearum* colony strain GZ3639 were inoculated into malt extract broth (Rodríguez et al., 2011) and allowed to grow for 10 days in baffled flasks at 28°C and 150 rpm in dark. Fungal mass was pelleted by centrifugation at 4,000 × g for 10 min at 4°C and the media was filter sterilized using a 0.22 μM syringe-driven filter (Millipore). The Fg-spent media was mixed with 10^3^ macroconidia per mL, to be used as the final inoculant for induction of *C. rosea* cultures.

The *Cr*-spent media extract (secretome) was prepared as follows. Four plugs of actively growing *C. rosea* ACM941 mycelia (harvested using the end of a 1 ml sterile pipette tip for consistency) were inoculated into 50 ml Czapek-Dox broth (HiMedia, USA), in 250 ml baffled flasks and incubated at 25°C an 180 rpm. At 21 days post-inoculation, fungal mass was pelleted by centrifugation at 4,000 × g for 10 min at 4°C and the medium was filter-sterilized using 0.22 μm syringe-driven filter (Millipore).

The treatment of *C. rosea* cultures with either MeOH (5 μL; solvent), DON (to a final concentration of 5 ppm, based on earlier similar studies (Kosawang et al., 2014), or *Fg*-spent media containing 10^3^ *F. graminearum* macroconidia per mL (final dilution 1:1,000), was completed at 5 days after initial inoculation with *C. rosea*, in 50 ml liquid Czapek-Dox broth (HiMedia, USA) at 25°C and 180 rpm. The treated cultures were allowed to grow under the same conditions for an additional 6 days and the media processed as described for un-treated cultures.

The *F. graminearum*-growth inhibition plate assay was carried out as follows. Different concentrations of filter-sterilized *Cr*-spent-media were spread on PDA plates (achieved by spreading different total volumes in the range of 0.375–2.25 ml) and allowed to dry in a laminar flow hood. Three actively growing *F. graminearum* strain GZ3639 agar plugs were subsequently placed on each plate at approximately equal distance from each other. Each filtrate concentration level was replicated three times, for a total of nine *Fusarium* colonies per experiment, and diameter of the fungus was measured at specified time points. In the case of treated *Cr*-spent media extracts, *F. graminearum* growth inhibition assays were conducted as described above by applying 750 μl of the treated *Cr*-spent media sterile filtrates to the PDA plates.

### *Clonostahcys rosea* ACM941 transcriptome profiling

Four plugs of actively growing *C. rosea* ACM941 mycelia were inoculated into nine 250 ml baffled flasks containing 50 ml Czapek-Dox broth (HiMedia, USA) and incubated at 25°C and 180 rpm for 5 days prior to treatment addition. As per Kosawang et al. (2014), either methanol (5 μL; solvent), DON (to a final concentration of 5 ppm), or *Fg*-spent media containing 10^3^ macroconidias per mL (final dilution 1:1,000), were added in triplicate and the fungus was allowed to grow for an additional 72 h before harvesting mycelia for RNA extraction. The mycelia were separated from the medium by centrifugation at 4,000 g for 10 min at 4°C, the fungal mass was flash frozen in liquid nitrogen, and genomic DNA-free high quality total RNA was extracted from each pellet using a combination of TRIzol® reagent (Thermo Fisher Scientific) and InviTrap® Spin Universal RNA mini Kit (Stratec molecular, Germany). Briefly, freeze-dried fungal mass was ground to a fine powder and homogenized in 1 ml TRIzol solution followed by genomic DNA removal and total RNA isolation using InviTrap spin columns following the manufacturer's protocol. After RNA concentration and purity was determined using a Nanodrop spectrophotometer ND-1000 (Thermo Scientific), its integrity was confirmed by agarose gel electrophoresis (Supplementary Figure 1). Total RNA (4 μg/sample) was shipped to the National Research Council of Canada, DNA Sequencing Technologies Facility (Saskatoon, Canada) where further quality check was performed using a BioAnalyzer followed by short cDNA fragment synthesis using TruSeq Stranded RNALT kit, and finally sequenced on an Illumina HiSeq 2500 platform according to the manufacturer's guidelines (Illumina, USA). Full RNAseq data is available from the NCBI (Bioproject ID = PRJNA420967) at the following link: http://www.ncbi.nlm.nih.gov/bioproject/420967.

### *De novo* assembly and sequence analysis

Low quality short reads, and adapter and other Illumina-specific sequences were filtered using Trimmomatic software v0.36.4 (http://www.usadellab.org/cms/index.php?page=trimmomatic) with the following modification to the default setting: leading low quality cutoff was 17; sliding window was 5 bp with a minimum average quality score of 20, and read length cutoff was 60 bp instead of 36 bp (Bolger et al., 2014). All 9 sets of cleaned paired-end *C. rosea* ACM941 data were assembled *de novo* using Trinity (v2.4) default settings with the addition of –jaccard_clip to minimize falsely fused transcripts. Reads were then mapped to assembled transcripts using the alignment-free tool Salmon (v.0.8.2) with default settings. For comparison, we also mapped our raw reads to *C. rosea* strain CBS125111 genomic sequence and annotations v1.0 retrieved from JGI (http://genome.jgi.doe.gov/pages/search-for-genes.jsf?organism=Cloro1; accessed on Dec 7th, 2016) and *C. rosea* IK726 putative transcripts (accessed on Sept 14, 2017) using the read mapper software STAR version 2.5.1 b (Dobin et al., 2013). Raw read counts per gene were extracted with the software HTSeq-count v0.6.0 (Anders et al., 2015) following procedures described previously (Pertea et al., 2015, 2016).

### Differential gene expression analysis and annotation

The DESeq2 based SARTools (v1.5.1) pipeline described by Varet et al. (2016) was adopted for differential analysis of mapped *C. rosea* ACM941 RNAseq count data. A BH *p*-value adjustment was performed (Benjamini and Hochberg, 1995; Benjamini and Yekutieli, 2001) and the false discovery rate (FDR) was set at *p* < 0.05. Coding sequences (CDS) of each gene were subtracted from the reference genome sequence with the following tools: bedopts v2.4.19 (Neph et al., 2012) and bedtools v2.26.0 (Quinlan and Hall, 2010). Blastx-fast (*E*-value < 10^−3^) module of BLAST2Go was used to align translated *C. rosea* ACM941 unigenes against the NR database (NCBI non-redundant protein library—last accessed August 10, 2017) to improve annotation coverage. Then different modules of the BLAST2Go software were used to run blast analyses, retrieve gene ontology (GO) terms and enzyme codes associated with each transcript, annotation, and mapping to KEGG pathway database and for graphical presentation of annotation results.

### Quantitative PCR validation of RNAseq expression results

To validate RNAseq gene expression results, primers (Table 1, primer set I) were designed for the following ten candidates selected based on the following criteria: Actin: housekeeping gene (for normalization); PKS1: the highest up-regulated gene in Fg-spent medium vs. MeOH library; sulfate permease: the highest up-regulated gene in DON vs. MeOH library and also one of the most up-regulated genes in Fg-spent medium vs. MeOH library; five ABC transporters: up-regulated in Fg-spent medium vs. MeOH library and/or DON vs. MeOH library; sterol 24-C-methyltransferase: down-regulated in Fg-spent medium vs. MeOH library and unchanged in DON vs. MeOH library; and hydrolase-1: unchanged in both libraries. Gene-specific primers (see Table 1) used in quantitative PCR (RT-qPCR) experiments were designed using the IDT primer quest software (https://www.idtdna.com/Primerquest/Home/Index) targeting 190–200 base-pairs (bp) fragment sizes. cDNA was synthesized from total RNA stored at −80°C (remaining from samples shipped for sequencing) using the 5X All-In-One RT MasterMix Kit (ABM, Inc., Canada). The relative transcript abundance of the selected genes was assessed by IQ™5 multicolor real-time PCR detection system (Bio-Rad, USA) using the EvaGreen Express 2X qPCR MasterMix (ABM, Inc., Canada) along with approximately 150 ng of cDNA template and 500 nM of each of the primers in a 20 μl reaction volume. The following program was used for RT-qPCR: 95°C for 1 min followed by 40 cycles of 5 s at 95°C and 15 s at 59°C. Normalized relative expression values (ΔΔC_T_) of the selected candidates were calculated using the formula 2^−ΔΔ*CT*^ (Livak and Schmittgen, 2001) using β-actin as a reference gene. Serial dilutions of cDNA samples were used to develop standard curves for primer sets to confirm their efficiency according to the Livak relative-gene expression quantification method.

**Table 1:** Primers used in this study.

**SET I (GENERAL VALIDATION)**		
Crosea_g5068	F: CCGTAGGAAATGAGGAGAAA	R: CAAACTCTGCCACACAATC
Crosea_g7462	F: GCAAATGCTCACGACTTTAT	R: GTACGATTCCCTCGGATTT
Crosea_g8930	F: CACCATTGCACATAGGTTATC	R: GCTCCTGTAAATCGGTATCA
Crosea_g10266	F: AGACCGGAGGTCATATACA	R: CCCAGCACAGGATCATAAA
Crosea_g6810	F: GGTCTCATCTTCGGAAACA	R: GATAGAACGCGGATCAAGTA
Crosea_g11869	F: CCTGCAGTTCCGGTTTAT	R: CATCACCTCCTCCTTATCATC
Crosea_g7	F: CGTCTTTGATGGGTTCTTTG	R: TGAACAGCATCTCGTAGTC
Crosea_g7906	F: CTTCTACGAGTACGGCTTC	R: CGGTGAACTTGACGATCT
Crosea_g5853	F: GTACAACGACCTCGACAA	R: CTTGCAGTTGAGCAGATTC
Crosea_g22712	F: GCGGTAGGACTGTGTATTT	R: TGGTACTCGGTACTCTCAA
**SET II (CLUSTER 2 VALIDATION)**		
Crosea_g12508	F: CCAAGACTTTACTTGGCATAAG	R: GGTGGTAATACAGAGAGTCAG
Crosea_g561	F: GGAGTATGTGAAGCGTCTATG	R: GTTTGAATATCCTCTGGCTACA
Crosea_g17059	F: ATCAAATCTGGGAATGCAAAG	R: CCACTCATCTGTCTTGTAGTT
Crosea_g5898	F: CTCCCGATTTATCTGCTAAGA	R: TGGCCACAGAAAGATACATAG
Crosea_g1996	F: TATTCAGTGGTCTGGTACAATC	R: CGAGTATGTAGGGAAGAACAC
Crosea_g8930	F: CACCATTGCACATAGGTTATC	R: GCTCCTGTAAATCGGTATCA
**SET III (CLUSTER 3 VALIDATION)**		
Crosea_g12258	F: AGGCTCTATCGAAGTCAATG	R: GGTGTTCCTAGTAGGTGATG
Crosea_g12255	F: GAAGTTCCGAGTTTGGTAATC	R: GATATGGAGGTCCAAGAGTT
Crosea_g12256	F: ACATCCTCACGATGCTTATG	R: CTGGTGTGATGTCGATACTG
Crosea_g12257	F: GCCCAACTTTGTCAGATAAC	R: CAGTTGGTACAAGGTGTTTG
Crosea_g12260	F: GACGGCAAAGTAGTAGAAGAA	R: TGCTGTCTGGATCATCTATTG
Crosea_g12260	F: CCGTGTCGAGAATTCAACTA	R: CCCAGTAGTCAATGCCATAA
Crosea_g12254	F: GTCTTTAACTTGGGCGATTG	R: TGCAGATTTCTCTAGCCTTT
**SET IV (CLUSTER 1 VALIDATION)**		
Crosea_g17059	F: CGTTCCAATGGAGACATAGA	R: GTCCAGATCAGAGTGGTATTC
Crosea_g15166	F: CCCAGAGGCATTAACTCTAA	R: CCGTTTCCGCAAATAGATAC
Crosea_g3952	F: GACAATCCTGCAACACCTTATC	R: GATGGGAGATAGGCCAGAATG
Crosea_g5068	F: CCGTAGGAAATGAGGAGAAA	R: CAAACTCTGCCACACAATC
Crosea_g7	F: CGTCTTTGATGGGTTCTTTG	R: TGAACAGCATCTCGTAGTC

### Differentially regulated gene cluster mining and validation

Gene/transcript identifiers of *C. rosea* strain CBS125111 were assigned to coding sequences based on their relative locations on its genomic sequence organized in scaffolds (unpublished data kindly made available by Joey Spatafora, Kathryn Bushley and JGI). Thus gene cluster members residing on the leading strand will have consecutive identification numbers but those residing on the trailing strand will have different but also consecutive numbers. Since secondary metabolite biosynthesis-related genes are typically organized into gene clusters, we took advantage of the assigned transcript identifiers to manually screen the relative expression library generated based on strain CBS125111 genomic sequence to identify differentially regulated gene clusters. First, a *Fusarium*-spent medium-induced *C. rosea* ACM941 expression library was sorted according to transcript identification number and the expression levels of genes surrounding up-regulated putative secondary metabolite genes were studied. If three or more consecutive genes surrounding the putative secondary metabolite gene were up-regulated, their sequences were retrieved and blasted against the reference genome using the blastn tool in the JGI database MycoCosm portal. Second, genomic sequences, including flanking regions of the first and last up-regulated genes, were retrieved and aligned against the entire CDS library using the blastn suite of NCBI to acquire gene cluster members residing on the opposite strand. Once all up-regulated genes and their flanking sequences were obtained, the complete cluster sequence was submitted to the fungal version of AntiSMASH online secondary metabolite prediction tool for verification and prediction (Medema et al., 2011). The differential expression results of gene cluster members were then validated using RT-qPCR primers (Table 1 primer sets II–IV) following the procedure described above.

### Statistical analysis

All experiments were replicated at least three times, and one-way ANOVA and multiple mean separation modules of the GraphPad prism (version 7.0, Graphpad Software, San Diego, CA) were used to calculate statistical differences and compare the means, respectively.

## Results

### *C. rosea* ACM941 culture filtrate inhibits *fusarium* growth

**Figure 1:**
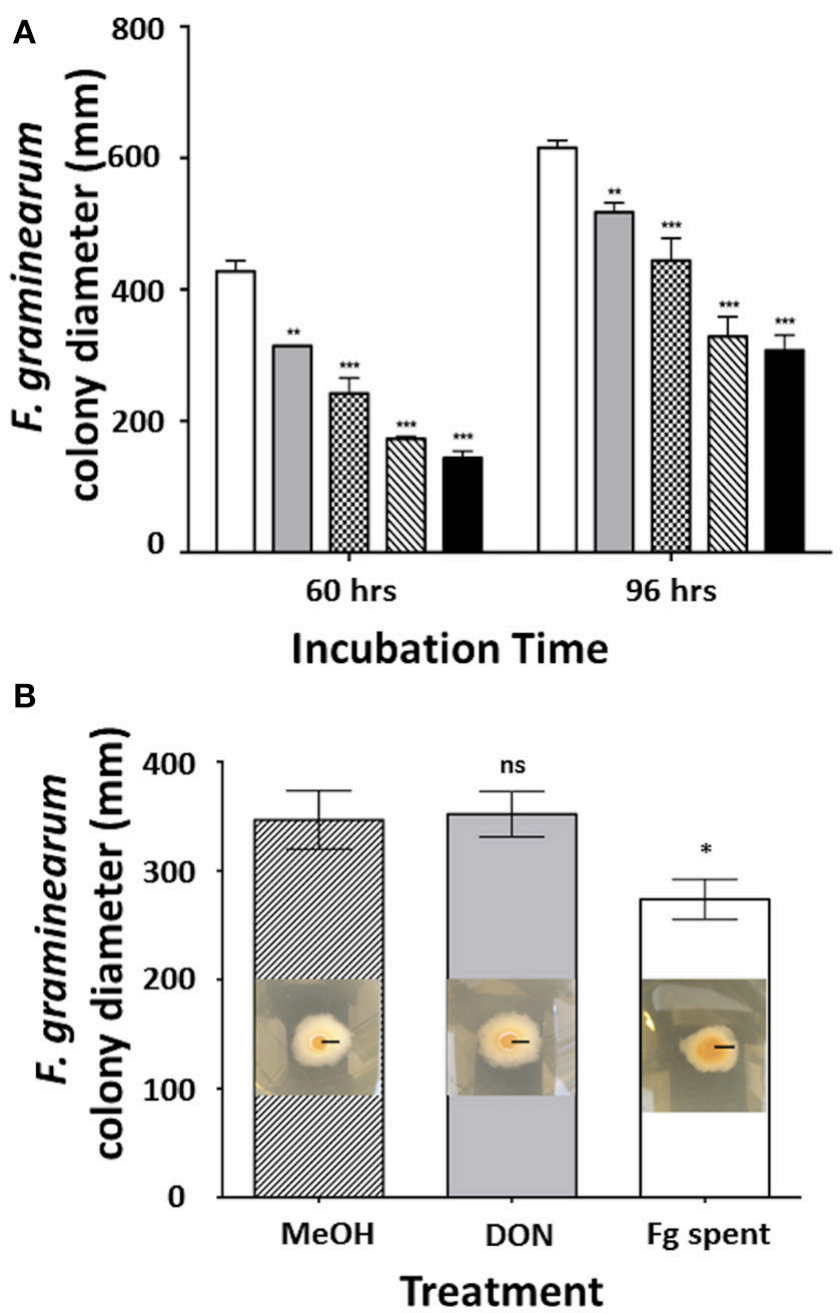
Effect of filter-sterilized secretomes on the *C. rosea AMC941*-*F. graminearum* interaction. **(A)** Effect of increasing concentration of filter-sterilized *C. rosea* ACM941 culture broth (representing its secretome) on *F. graminearum* colony size after 60 and 96 h incubation. Bars represent: white: *F. graminearum* grown on PDA plate overlaid with 2.25 ml of Czapek Dox broth (control); and gray, plaid, diagonal stripe, black: *F. graminearum* grown on PDA plates overlaid with 375, 750, 1,500, and 2,250 μl of filter-sterilized *C. rosea-*spent media, respectively. Asterisks indicate level of significance at *p* < 0.001 and error bars indicate standard error (*n* = 3). **(B)** Effect of DON and *Fusarium-*spent media treatment (applied during the production of *C. rosea* ACM941 spent-media) on the growth-inhibitory activity of *C. rosea* ACM941 spent-media. Shown are representative images of *F. graminearum* grown on PDA plates overlaid with sterilized *C. rosea*-spent-media obtained after growth in the presence of (i) methanol, (ii) 5 ppm DON, and (iii) 1,000 × diluted *F. graminearum* spent media containing live macroconidia. Black bar indicates 1 cm. These are embedded in a quantitative plot of the data obtained. Asterisks indicate level of significance at *p* < 0.001 and error bars indicate standard error (*n* = 3).

Previous reports showed that *C. rosea* strain IK726 secretes heat-stable metabolites that inhibit the growth of *F. graminearum in vitro* (Dubey et al., 2014). In order to test whether the *C. rosea* strain ACM941 mediates a similar effect, *F. graminearum* strain GZ3639 growth inhibition assays were completed, showing that it does also secrete antifungal metabolites when grown in liquid culture (Figure 1A). Indeed after 60 h incubation, reductions in *F. graminearum* radial growth ranged from 27 to 67% with increasing ACM941 spent media filtrate applications, compared to *F. graminearum* grown in the absence of the filtrate. Longer incubations, to 96 h, still showed effects, with radial growth reduction ranging from 16 to 50%. On the flip side, the effects of *F. graminearum*-spent media, compared to exogenous DON and a methanol control, were also investigated to assess whether the growth inhibitory effects of the *C. rosea* ACM941 spent-media filtrate might be affected by factors secreted by the pathogen. Toward this, either *F*. *graminearum* culture filtrates that contained live macroconidias, (1:1,000 overall dilutions) were added to growing *C. rosea* cultures and the *C. rosea* culture filtrate harvested after 6 days of induction. Subsequent application of each of these *C. rosea* culture filtrates back to a *Fusarium* growth inhibition assay, demonstrated a modest induction of the *C. rosea* culture filtrate growth-inhibition effect, compared to either the 5 ppm DON or MeOH treatment and the methanol control (Figure 1B). No significant difference was ever detected between the MeOH treated samples and an “un-treated” control sample (data not shown).

### *C. rosea* strain ACM941 sequence analysis

On the basis of the above growth responses, a global expression profile of *C. rosea* strain ACM941 was developed for the three treatment conditions, to enable investigation of the molecular responses leading to the observed effects. The RNAseq library (see methods section for link to full RNAseq data set) was developed from high quality total RNA isolated from *C. rosea* mycelial pellets treated for 3 days with methanol, 5 ppm DON and 1:1,000 diluted *F. graminearum* culture filtrates containing live macroconidias, respectively. The RNA was isolated at day 3 because genetic effects leading to the observed physical differences at day 6 are expected to be initiated earlier, to account for the time needed for metabolites to be produced, secreted and accumulate to levels that are sufficient to elicit growth inhibition against *F. graminearum* (Kosawang et al., 2014). A total of 220,828,191 raw reads were obtained from the nine samples sequenced, using an Illumina HiSeq 2500 platform. Of these, 203,792,968 were deemed to be high quality clean reads after removing low quality sequences and adapters. Notably, >92% of the reads were successfully mapped to the *C. rosea* IK726 transcript library (see below) using TopHat, implying little if any contamination of the RNA by F. graminearum. The average GC content was 52.06%. All processed reads from the nine samples were assembled, using Trinity (v2.4) software, to 78,959 transcripts which were further processed to 24,112 unigenes, comprising 43,671,104 bp with an average contig length of 1,673 bp and N_50_ value of 3,451 bp. According to the “blastn task-megablast” module of the Blast2Go library comparison results, approximately 81% (15,423) of the *C. rosea* strain CSB125111 genes found at least one hit in our *de novo* assembled unigenes library with >74% sequence identity at *E*-value less than 10^−51^. In contrast, nearly 89.2% (12,719) of *C. rosea* strain IK726 transcripts (14,268) had one or more homeologous unigenes in our Trinity assembled library with >73% sequence identity and *E*-value less than 10^−51^. We also compared CSB125111 genes against all putative IK726 transcripts by megablast analysis and only 64.1% (12,229) of them identified one or more homolog hits with >74% sequence identity at *E*-value less than 10^−51^. These results show that *C. rosea* strain ACM941 is genetically closer to strain IK726 than CSB125111.

To validate the assembly, raw reads from a representative replicate of our RNAseq data were also assembled *de novo* using the CLC-Genomics work bench software *de novo* assembly module (Qiagen, USA), as per the default parameters with the minimum read cut-off value set to 60 bp. The assembly generated 29,145 contigs with an average contig length of 1,261 bp and an N_50_ value of 1,973 containing 36,758,016 bp. Subsequent megablast analysis using CBS125111 genes as query identified only 14,302 genes (75%) to have at least one homolog contig in the CLC assembled library compared to 81% for Trinity assembled unigenes. In addition, while 13,480 of the 19,064 CBS125111 genes had single hits when blasted against the Trinity assembled unigenes, only 9,674 of them had single hits when blasted against CLC assembled contigs, suggesting a high rate of redundancy in the CLC assembled library. Thus, further analysis was performed on the Trinity-assembled unigenes going forward.

### Functional annotation of the *C. rosea* strain ACM941 transcriptome

A blast analysis of the *C. rosea* ACM941 transcript library against the NR (non-redundant) database identified hits for 82.5% of the unigenes, 83 and 59% of which had gene ontology (GO) mapping and annotation results available, respectively. The sequence similarity distribution showed that 88% of the blast hits have >50% sequence similarity level while *E*-value distribution showed that 73.5% of the unigenes had high homology with an *E*-value less than 10^−50^. The remaining 17.5% unigenes that did not yield blast hits might represent genes that are yet to be identified, genes that are specific to *C. rosea*, or, alternatively, false positives.

**Figure 2:**
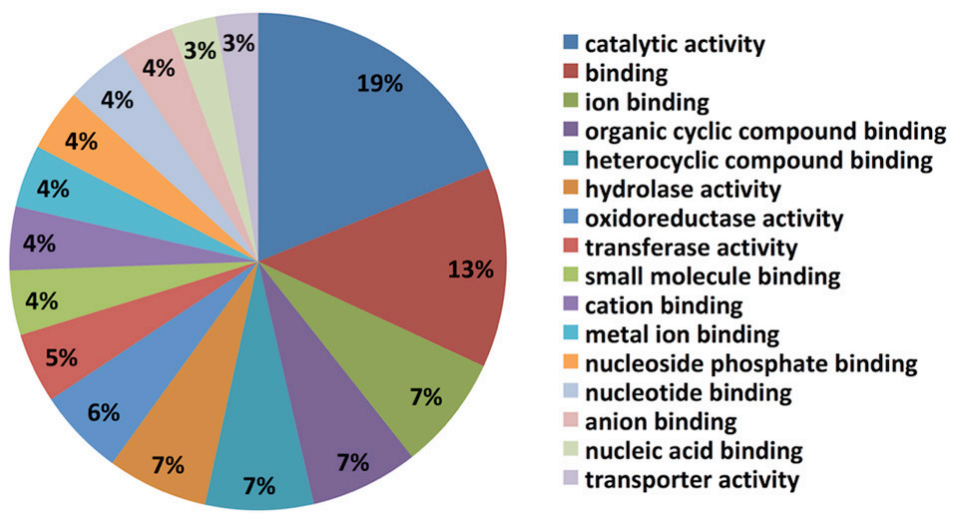
Gene ontology classification results of Trinity assembled *C. rosea* AMC941 unigenes. Molecular level classification of Trinity assembled unigene GO terms. The percentages represent the ratio of major GO terms assigned to our unigenes.

The top blast hit species, with 13% of transcripts showing homology, is the prolific secondary metabolite-producing fungus *Acremonium chrysogenum*, followed by *Neonectria ditissima* (5.9%), *Nectria haematococca* mpVI 77-13-4, a teleomorph of the destructive plant pathogen *F. solani* (5.7% homology), *Purpureocillium lilacinum* (2.4%) and *Tolypocladium ophioglossoides* CBS 100239 (2.3%) etc. (Supplementary Table 1). It is noteworthy that most of the top hit species are either endophytes and/or micoparasites regarded for their prolific secondary metabolite biosynthesis capability and their ability to be used as bio-control agents. Molecular level classification of GO terms showed the majority of *C. rosea* ACM941 unigenes (7%) encode for proteins with ion binding function, followed by organic and heterocyclic compound binding, each also accounting for 7% of the annotated library. Genes encoding for hydrolase and oxidoreductase enzymes represented 7 and 6% of the libraries, respectively, while 5 and 4% of them were annotated to have transferase activity and small molecule binding roles, respectively (Figure 2).

### Differential gene expression analysis of *C. rosea* strain ACM941 treated with DON or *fusarium* spent medium

**Figure 3:**
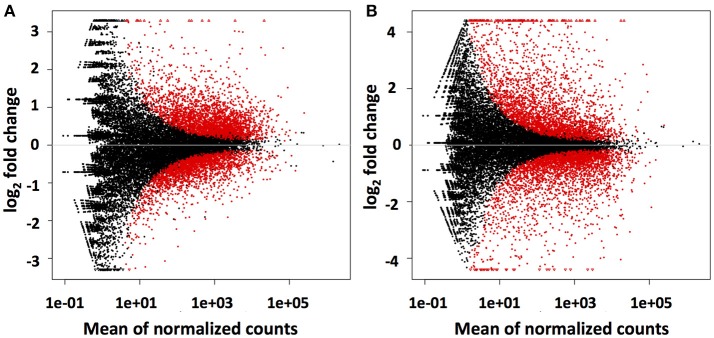
Differential regulation of Trinity assembled AMC941 *C. rosea* unigenes. Volcano plots of differential gene expression relative to meOH treated *C. rosea* for, **(A)** 5 ppm DON treated and **(B)** *Fusarium*-spent media (containing live macroconidias) treated *C. rosea*. Volcano plots were obtained using DESeq2 software (Varet et al., 2016).

Mapping of the raw RNAseq expression data against the Trinity-derived unigene library showed that a total of 5,605 (23%) and 6,285 (26%) of the 24,112 *C. rosea* unigenes were differentially regulated by DON and *Fusarium-*spent medium treatment, respectively (Figure 3). In both treatment cases approximately half of the differentially regulated transcripts were up-regulated, at 3,167 and 3,121 respectively. Based on the premise that bio-control is likely arising due to induction of the biosynthesis and secretion of antifungal-secondary metabolites from *C. rosea* in response to *F. graminearum*, the remainder of this report is focussed on up-regulated unigenes putatively involved in these processes.

**Figure 4:**
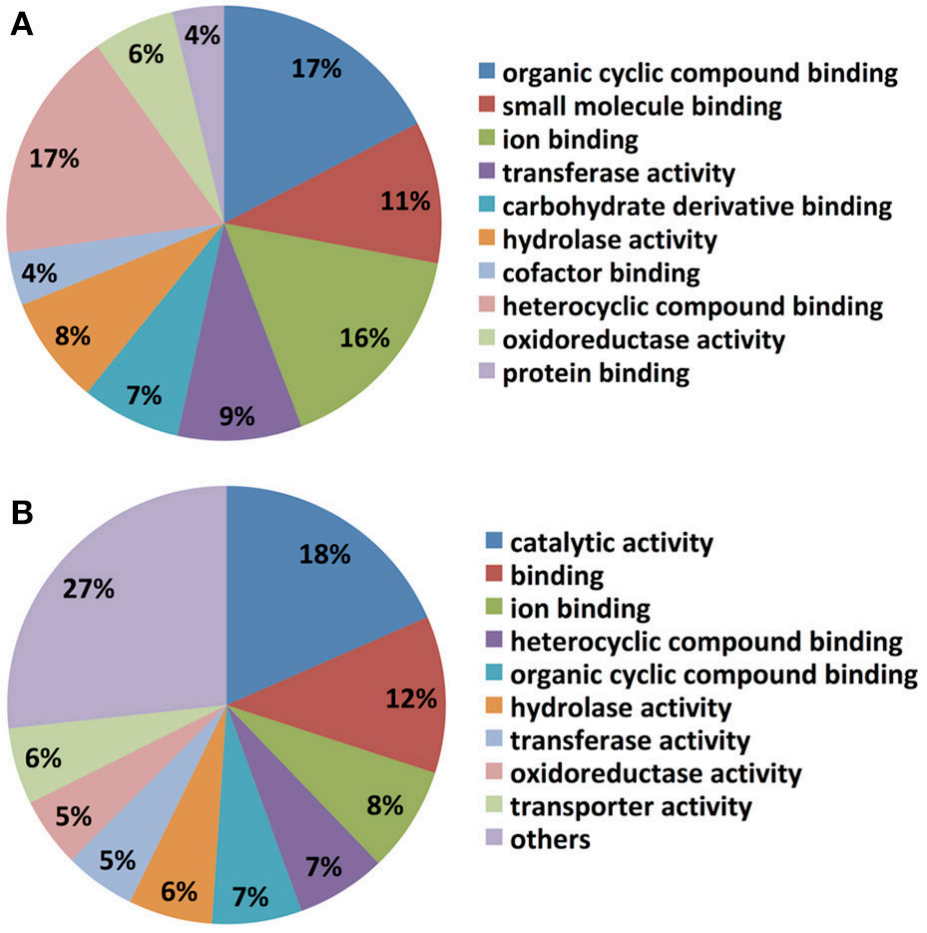
Molecular level classification of up-regulated hits in the AMC941 unigene library. Up-regulated gene hits were evaluated by BlasttoGO, and molecular level classification assignments relative to MeOH treated *C. rosea*, obtained for **(A)** *C. rosea* treated with 5 ppm DON and **(B)** *C. rosea* treated with *Fusarium-*spent media (containing live macroconidias), both relative to MeOH treated *C. rosea*.

Not surprisingly, most unigenes up-regulated by DON treatment were also up-regulated by *Fusarium* spent medium containing live macroconidias, implying overlap of *C. rosea's* responses to the two treatments. In particular, unigenes putatively encoding enzymes and transporters involved in amino acid biosynthesis and transport, as well as fatty acid biosynthesis were found to be up-regulated by both treatments, based on KEGG mapping of GO terms (e.g., Supplementary Figure 1). Notably, two transcripts with homology to enzymes involved in long chain fatty acid breakdown to acetyl-CoA including a putative acyl-CoA synthetase (Crosea_g3952 or Contig_740), which was up-regulated 2.6- and 865-fold, while a putative peroxisomal 2,4-dienoyl-CoA reductase (Crosea_g15166 or Contig_17275) was up-regulated 2.3- and 71-fold, both by DON and *Fusarium-*spent medium treatments respectively. Molecular level functional classification of GO terms also showed that both libraries are dominated by cyclic compound- and ion- binding proteins (Figure 4). However, certain differences in the expression profiles for DON and *Fusarium*-spent medium treatment were noted, particularly in terms of representation and expression levels of unigenes putatively encoding fungal secondary metabolite biosynthetic enzymes. Indeed the single most highly represented GO term in the *Fusarium*-spent media-treated *C. rosea* up-regulated library was “catalytic activity” (18%), a term that did not arise in the DON-treated sample unigenes GO annotations. In addition, transporter protein-encoding unigenes accounted for 6% of the *Fusarium* spent medium treated library, while enrichment of cofactor, protein and carbohydrate derivatives binding proteins are highlighted in the DON library.

### Differential expression result validation

**Figure 5:**
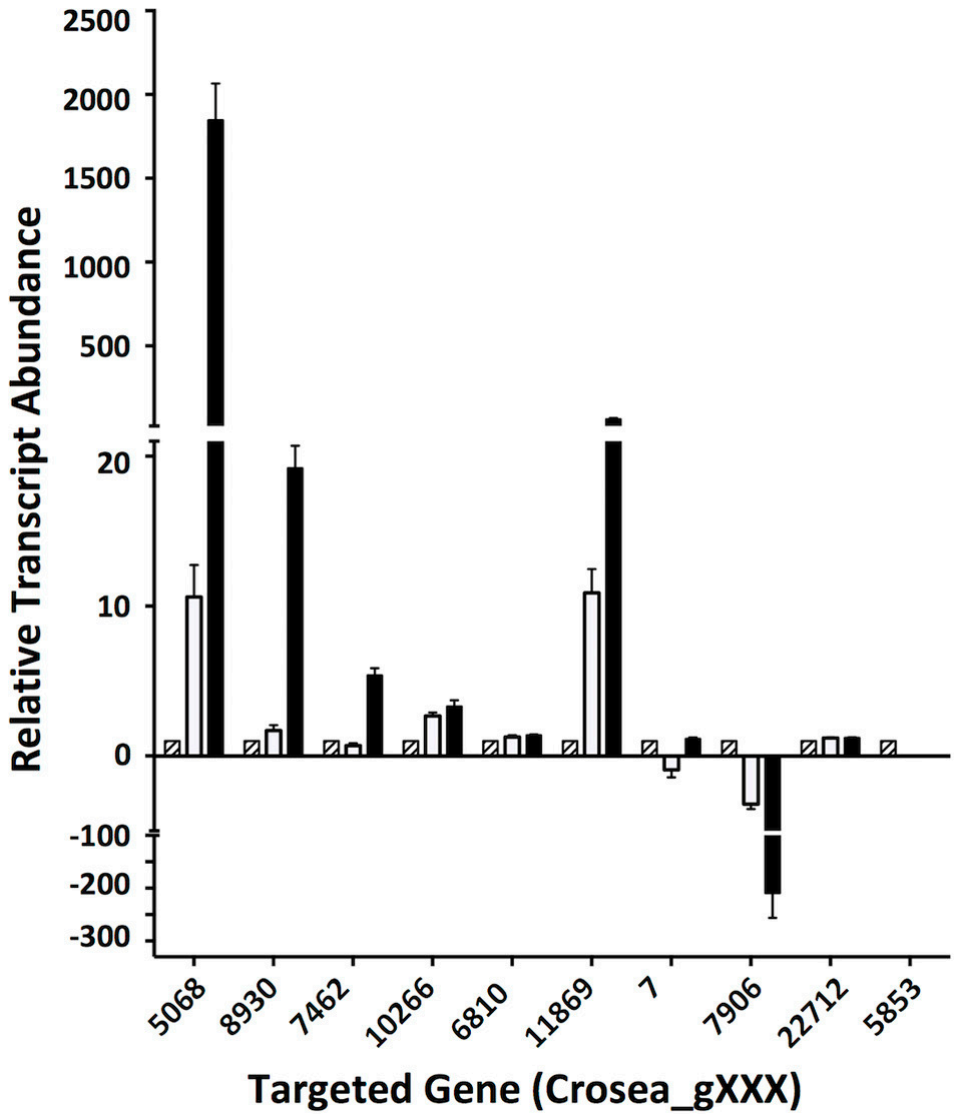
Validation of differentially regulated AMC941 *C. rosea* transcripts using RT-qPCR. Crosea_g5068: polyketide synthase; Crosea_g8930: multidrug resistance transporter-1, Crosea_g7462: multidrug resistance transporter-4; Crosea_g10266: multidrug resistance transporter-3; Crosea_g6810: multidrug resistance transporter-2; Crosea_g11869: sulfate permease, Crosea_g7: Hydrolase; Crosea_g7906: sterol methyltransferase; Crosea_g22712: pleiotropic drug resistance transporter; Crosea_g5853: Cytochrome p450. Black bars represent the data obtained for the *F. graminearum* spent-media treated samples, white bars represent the data obtained for the DON-treated samples and the hatched bars represent the data obtained for the MeOH-treated samples. Error bars indicate standard error (*n* = 9).

In line with our main objective of identifying up-regulated putative secondary metabolite biosynthesis- or transport- related genes, a selection of nine ACM941 Trinity-assembled unigenes were selected for further analysis on the basis of their expression levels and possible role in antifungal or antibiotic compound biosynthesis (see genes marked with hastags (#) in Table 2). These include highly induced genes annotated as polyketide synthetases (PKSs), non-ribosomal peptide synthetases (NRPSs) and ABC transporters. For each of these nine gene targets, quantification of expression levels by RT-qPCR results using *de novo* prepared samples, showed levels mirroring those obtained from RNAseq analysis (Figure 5 and genes with hashtags (#) in Table 2). For example, the average PKS1 (Crosea_g5068) expression in RNAseq analysis using the Trinity-assemble unigenes as reference was 2,387 fold-induced compared to MeOH control (Table 2), while its average relative expression for a total of nine replicates using RT-qPCR was calculated as 1,846 ± 660-fold (Figure 5). This analysis also highlights that not only does the *Fusarium*-spent media have a more dramatic effect on the overall level of induction compared to DON-alone, but that it also induces the expression of genes not otherwise affected by DON-alone (Figure 5).

**Table 2:** Differential expression of cluster members and selected genes in all expression libraries.

	**CBS125111 extracted transcript library ID**	***A***	***B***	**IK726-trasncript library ID**	***A***	***B***	**ACM941 Trinity assembled library ID**	***A***	***B***	**ACM941 CLC- assembled library ID**	***A***	***B***	**Homology description**
Cluster 1	XLOC_018256	2	2.38	BN869_T0007384_1	1.6	2.3	Crosea_g3331	1.7	2.4	Contig_6710	1.8	2.5	Transcription factor
	XLOC_018258	NA	79.81	BN869_T0007382_1	2.2	64.6	Crosea_g15166	2.3	68	Contig_17275	3.8	70.9	Dienoyl CoA-ligase
	XLOC_018259^*^^#^^&^	NA	2591	BN869_T00007381_1	63.5	2933	Crosea_g5068	62.6	2840	Contig_1989	49.6	2387	Polyketide synthase
	XLOC_017877^*^^#^	4	5.9	BN869_T00007385_1	5.7	5.6	Crosea_g7	5.6	5.3	Contig_10313	5.7	5.6	Hydrolase
	XLOC_017878	NA	847.9	BN869_T00007380_1	32	1283	Crosea_g3952	2.5	73.6	Contig_740	24.8	865.3	CoA-ligase
Cluster 2	XLOC_002182	NA	18.3	BN869_T00002234_1	4.6	17.8	Crosea_g17059	4.8	18.3	Contig_3577	4.6	18.4	Oxidoreductase
	XLOC_002183^&^	NA	22.63	NA	NA	NA	Crosea_g561	2.5	16.7	Contig_1783	2.6	19.7	Non-ribosomal peptide synthase-short
	XLOC_002184^&^	NA	2202	NA	NA	NA	Crosea_g12508	2	187	Contig_1528	11.8	1942	Polyketide synthase
	XLOC_002457	NA	7.2	BN869_T00002236_1	1.6	7	Crosea_g5898	1.7	7.1	Contig_1873	2.5	12	UDP-transferase
	XLOC_002458	NA	11.93	BN869_T00002232_1	3.4	12	Crosea_g1996	1.73	3.26	Contig_11538	1.9	4.7	UDP-transferase
	XLOC_002460^*^^#^^&^	NA	4.81	NA	NA	NA	Crosea_g8930	3.5	11.8	Contig_2435	3.2	11.5	Multidrug resistance transporter
Cluster 3	XLOC_008927^&^	NA	2.27	BN869_T00012929_1	1.8	2.6	Crosea_g12260	1.9	3.9	Contig_1695	1.8	2.57	Multidrug resistance transporter/FsqE
	XLOC_008928	NA	12.77	BN869_T00012930_1	1.6	12.7	Crosea_g12260	1.9	3.9	Contig_1737	1.6	12	ATP grasp/fsqD
	XLOC_008929	NA	58	BN869_T00012931_1	1.8	56.6	Crosea_g12254	1.9	58.7	Contig_2615	1.8	54	N-Methyl Transferase/FsqC
	XLOC_009016	NA	11.98	BN869_T00012928_1	1.6	11.1	Crosea_g12258	1.7	10.6	Contig_24611	1.6	11	Phenol-monooxygenase/fsqG
	XLOC_009017	NA	45.05	NA	NA	NA	Crosea_g12255	2.1	44.8	Contig_6597	2	44.2	Amino acid oxidase/FsqB
	XLOC_009018	NA	7.46	NA	NA	NA	Crosea_g12256	1.2	5.6	Contig_7067	1.3	8	Transcription Factor/FsqA
	XLOC_009019^&^	NA	42.72	NA	NA	NA	Crosea_g12257	2.2	44.9	Contig_879	1.9	41.2	Non-ribosomal peptide synthase-short/FsqF
RT-qPCR validation targets not located in clusters	XLOC_019006^#^^&^	1.5	1.8	BN869_T00004618_1	2.7	2.5	Crosea_g6810	2.2	3.2	Contig_8124	2.6	2.4	Multidrug resistance transporter
	XLOC_001628^#^^&^	2	1.9	BN869_T00013092_1	2.3	2	Crosea_g10266	2.9	3.7	Contig_1568	2.1	2.1	Multidrug resistance transporter
	XLOC_016258^#^	2.1	0	BN869_T00005711_1	2.3	0.014	Crosea_g7906	2.3	−5.39	Contig_25197	2.1	−154	Sterol methyltransferase
	XLOC_018801^#^^&^	NA	39.7	BN869_T00008748_1	2	4.9	Crosea_g7462	2	5	Contig_3181	1.8	4.7	Multidrug resistance transporter
	XLOC_017405^#^	81.8	184.4	BN869_T00004217_1	10.9	30.4	Crosea_g11869	1.6	1.1	Contig_1856	3.5	8.7	Sulfate permease
	XLOC_002915^#^^&^	NA	NA	BN869_T00005295_1	0.5	1.8	Crosea_g22712	0.6	1.8	Contig_6478	-2.5	1.6	Pleiotropic drug resistance transporter
	XLOC_011084^#^	NA	NA	NA	NA	NA	Crosea_g5853	1.2	−3	Contig_4631	1.1	−4	Cytochrome p450

A, expression in response to DON treatment relative to methanol; B, expression in response to Fusarium-spent media containing live macroconidias treatment relative to methanol. NA indicates not available.

* indicates candidate is a member of top 20 hits sorted based on CBS12511 RNAseq analysis.

# indicates the nine candidates used in validation experiments.

& *indicates candidates selected as anchor genes. Remaining members of the top 20 hit list, not located in clusters, are included in Supplementary Table 2*.

Another approach applied to validate the differential expression analysis results obtained from mapping expression against the ACM941 Trinity-assembled unigenes, was to map the raw RNAseq expression data against the ACM941 CLC-assembled unigenes, as well as against the two reference sequences: strain CBS125111 genome and IK726 transcripts. Using IK726 transcripts as a reference yielded 5,355 (37.5%) and 5,551 (38.9%) differentially regulated genes, for the DON and *Fusarium*-spent media treatments respectively; numbers that are consistent with those obtained using the ACM941 Trinity-unigene assembly reference. In contrast, when the CBS125111 (a more distantly related *C. rosea* species) genome was used as the reference, the total number of identified genes differentially regulated by DON and *Fusarium-*spent media treatment were significantly lower, at only 977 (5.2%) and 1,195 (6.3%), respectively. The reference-library dependent similarities and differences could be due to either (i) over-representation of up-regulated unigenes in the *de novo* assembled library or (ii) the genetic distance between the strains. Indeed, upon cloning and sequencing several genes (totaling >14 kb coverage) from strain ACM941, using primers designed based on IK726 transcripts, sequences with only a few bp differences between the two organisms were obtained, emphasizing their close relation. On this basis, the number of differentially regulated transcripts derived by mapping against the ACM941 Trinity-unigene assembly and the IK726 transcripts are considered quite realistic, and are also similar to values reported previously, where 6,890 (34.8%) unigenes were differentially regulated during *C. rosea* strain 67-1 interactions with *S. sclerotiorum* sclerotia (Sun et al., 2015).

At a more detailed level, the top 20 up- or down-regulated unigenes (based on expression observed upon mapping the RNAseq expression data against the Trinity-assembly reference; (Supplementary Table 2 and asterisks (^*^) in Table 2) and those genes targeted for RT-qPCR validation (see hastags (#) in Table 2), were then compared across the four different RNAseq expression data mapping libraries (i.e., using either Trinity-, CLC-, IK726- or CBS125111 databases as reference). In most cases the genes were similarly regulated across all libraries. The only exception was a lack of some highly-up-regulated unigenes when *C. rosea* IK726 putative transcripts were used as a reference. For example, the corresponding transcript for the second top hit that was annotated as a polyketide synthetase (Crosea_g12508 or Contig_1528) was absent in the IK726 transcript library. However, blast alignment of the unigene against the IK726 genome identified a hit with 100% identity, suggesting that the putative transcript library does not contain all possible genes in the genome. As such transcripts missing from the *C. rosea* IK726 putative transcripts library were included as extracted from the genomic sequence, for final analysis, ultimately showing similar responses in the differential expression result regardless of the reference used.

### Differentially expressed gene clusters in *C. rosea* strain ACM941

Up-regulation of unigenes annotated to be homologs of secondary metabolism-related biosynthetic genes, combined with the fact that secondary metabolite biosynthesis-related genes are often clustered in fungi, prompted us to study the expression patterns of genes adjacent to selected “anchor” secondary metabolite biosynthesis-related genes (see genes marker with ampersands (&) in Table 2). This analysis was based on the RNAseq analysis result deduced from the *C. rosea* strain CBS125111 reference genome and *C. rosea* IK726 putative transcripts, because their transcript IDs reflect their relative genomic locations. As expected, in a number of instances, genes with numbers consecutive to the “anchor” genes were also found to be up-regulated (Table 2). Blasting all up-regulated genes with consecutive IDs against the genomic sequence of *C. rosea* strain CBS125111 using the BLAST tool of JGI, mapped them into three genomic locations revealing that a number of the candidates identified as “top 20 expressing” or “targets for RT-pPCR validation” earlier, are part of three up-regulated putative gene clusters.

#### Cluster 1

**Figure 6:**
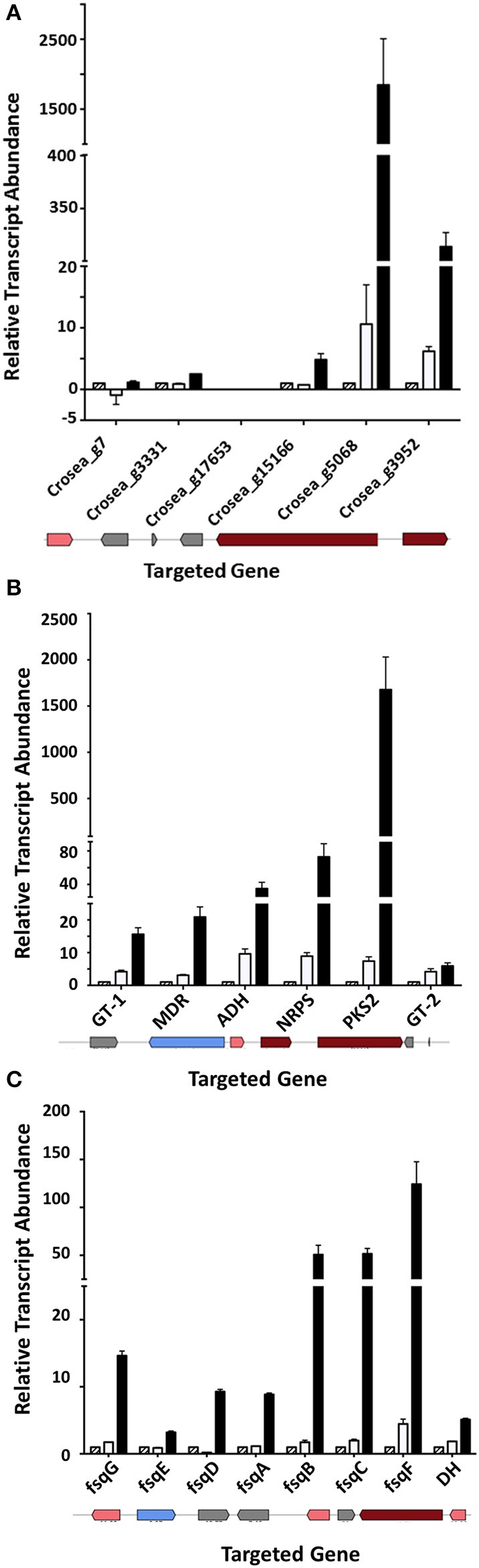
Up-regulated gene clusters in *Fusarium*-spent media treated AMC941 *C. rosea* library and their validation. **(A)** Genomic organization (bottom) and RT-qPCR validation of a putative cluster possibly involved in precursor biosynthesis for polyketide synthetases (Cluster 1). **(B)** Genomic organization (bottom) and RT-qPCR validation of a putative TMC-151 family biosynthetic gene cluster (Cluster 2). **(C)** Genomic organization (bottom) and RT-qPCR validation of a putative isoquinoline alkaloid gene cluster (Cluster 3). ADH, alcohol, dehydrogenase homolog (Crosea_g10759); D.E., RNAseq differential expression; DH, dehydrogenase; fsqA (Crosea_g12256): Zn(II)_2_Cys_6_-type transcription factor; fsqB (Crosea_g12255): fructosyl amino acid oxidase; fsqC (Crosea_g12254), N-methyltransferase; fsqD (Crosea_g12260), ATP-grasp enzyme; fsqE (Crosea_g12260), MDR-type ABC transporter; fsqF (Crosea_g12257), non-ribosomal peptide synthase; fsqG (Crosea_g12258), phenol 2-monooxygenase; GT-1 (Crosea_g5898), UDP-transferase; GT-2 (Crosea_g1996), UDP-transferase; MDR (Crosea_g8930): multidrug resistance transporter; NRPS (Crosea_g561), non-ribosomal peptide synthase-short and PKS2 (Crosea_12508) L polyketide synthase. DH was not included in the isoquinoline alkaloid Fumisoquin biosynthetic gene cluster in *A. fumigatus* (Baccile et al., 2016) but resides next to NRPS and up-regulated in *C. rosea*. In all cases black bars represent the data obtained for the *F. graminearum* spent-media treated samples, white bars represent the data obtained for the DON-treated samples and the hatched bars represent the data obtained for the MeOH-treated samples. Error bars indicate standard error (*n* = 3).

Three genes adjacent to one of the most highly up-regulated unigenes, Crosea_g5068, putatively encoding a PKS, were also up-regulated several fold in both Trinity and CLC-assembled libraries (Table 2). These include unigenes Crosea_g3331 putatively encoding a transcription factor, Crosea_g15166 with homology to peroxisomal 2,4-dienoyl-CoA ligase and Crosea_g3952 with homology to acyl-CoA synthase, also known as a long chain fatty acid CoA-ligase. The expression pattern of these unigenes obtained from RNAseq analysis was further validated using RT-qPCR (Figure 6A). The two adjacent genes encoding for acyl-CoA synthase and peroxisomal 2,4-dienoyl-CoA ligase are putatively involved in long chain fatty acid breakdown to acetyl-CoA, and are supposedly involved in precursor biosynthesis for polyketide synthetases (Poirier et al., 2006).

#### Cluster 2

The second gene cluster was identified using the other highly induced PKS homolog, Crosea_g12508, as an “anchor” gene that was up-regulated in response to *Fusarium-*spent medium treatment. Five genes adjacent to this anchor were found to be up-regulated as well (Table 2), including an NRPS-like homolog (Crosea_g561) and an ABC transporter (Crosea_g8930). The three other adjacent up-regulated genes included two with homology to sugar-transferases (Crosea_g5898 and Crosea_g1996) and the third, putatively encoding for an alcohol dehydrogenase (Crosea_g17059). The expression pattern of these unigenes obtained from RNAseq analysis was further validated using RT-qPCR (Figure 6B). The chemical reactions putatively expected to be catalyzed by members of this cluster suggest a potential role in biosynthesis of *C. rosea*'s unique antifungal metabolite, TMC-151 (Okuda et al., 2000; Zhai et al., 2016), discussed further below.

#### Cluster 3

Finally blasting Crosea_g12257, up-regulated by *Fusarium-*spent medium treatment, against the NR database showed that it putatively encodes a short NRPS-like peptide. This was notable being that NRPS-like peptides have shown potential in catalyzing the synthesis of alkaloids in fungi (Macheleidt et al., 2015; Baccile et al., 2016). Using this unigene as a query in blast analysis against the Swiss-Prot library showed that it is homologous to the *Aspergillus fumigatus* Fumisoquin biosynthetic gene cluster core gene fsqF (Baccile et al., 2016). Fumisoquin is an isoquinoline alkaloid that requires the activity of an array of proteins and enzymes for its biosynthesis in *A. fumigatus*, including fsqA a Zn(II)_2_Cys_6_-type transcription factor; fsqB a fructosyl amino acid oxidase; fsqC an N-methyltransferase; fsqD an ATP-grasp enzyme; fsqE an MDR-type ABC transporter; fsqF a short NRPS-like peptide and fsqG a phenol 2-monooxygenase. Analyzing genes adjacent to Crosea_g12257 (CrfsqF from here on) highlighted six other up-regulated adjacent genes, with each one having homology to one protein comprising the *A. fumigatus* Fumisoquin biosynthetic cluster (Table 2). The expression patterns obtained from RNAseq analysis of all cluster member unigenes was further validated using RT-qPCR (Figure 6C).

## Discussion

Understanding the molecular and biochemical basis of *C. rosea* mycoparasitism will enable development of more potent bio-control agents and identification of metabolites that could serve as novel lead compounds for development of effective, specific and thus more environmentally friendly fungicides against FHB. Secondary metabolites have long been suspected to mediate the bio-control ability and antifungal property of *C. rosea* against *Fusarium spp* (Rodríguez et al., 2011; Dubey et al., 2014). Supporting this hypothesis, recent large-scale sequencing results and comparative bioinformatics analyses of the *C. rosea* genome against other fungal species have highlighted a preferential enrichment of secondary metabolite biosynthetic genes and those involved in mycotoxin and fungicide tolerance (Karlsson et al., 2015). Therefore, it was not coincident that the top hit species in blast analysis reported here was *Achromiun chysogenum*, a fungus exploited industrially for its copious secondary metabolite biosynthetic ability, followed by other fungi known for their prolific secondary metabolite biosynthesis capacity and biocontrol ability. Together this emphasizes the likely role of secondary metabolites in the evolution of *C. rosea's* lifestyle as a mycoparasite. Elucidating the regulatory and structural genetics mediating the biosynthesis and localization of these secondary metabolites will be crucial to improving its bio-control ability. However, identifying the metabolites and linking them to the genetic loci responsible for their biosynthesis has remained a challenge, largely because routine laboratory conditions are not conducive to the expression of secondary metabolism-related genes, a phenomenon called “cryptic” or “silent” gene clustering.

Several strategies have been introduced to induce “cryptic” gene clusters in fermentation media, based on mimicking environmental cues and *in situ* microbial diversity, for example by adding filter-sterilized culture media of antagonistic or host microbe, and/or by co-cultivation (Marmann et al., 2014; Reen et al., 2015; Newman, 2016; Okada et al., 2017). This is particularly relevant to endophytic fungi cultured in fermentation media where, for example, growing the endophyte *Silybum marianum* with autoclaved leaves and plant extracts of the nominal host plant successfully reversed the loss of secondary metabolite biosynthetic ability of the fungus during sub-culturing (El-Elimat et al., 2014). Co-cultivation of endophytes with competing fungi strains has been routinely used to induce the expression of secondary metabolite related gene clusters and isolate new metabolites (described in detail in two recent reviews, Marmann et al., 2014; Netzker et al., 2015). As well, an RNAseq library containing secondary metabolite biosynthetic genes (PKs, NRPSs, and ABC transporters) was recently obtained by co-cultivating live sclerotia of *S. sclerotiorum* with *C. rosea* strain 67-1 in liquid medium (Sun et al., 2015).

*C. rosea* ACM941, like other strains including IK726 (Dubey et al., 2014), is an endophytic fungi genetically endowed with extensive secondary metabolite biosynthetic machinery and shown to secrete as yet unidentified metabolite(s) that inhibit the growth of *F. graminearum* strain GZ3639. Despite this, however, the EST library developed from *C. rosea* strain IK726 treated with 5 ppm DON in liquid medium did not show up-regulation of secondary metabolite biosynthesis or transport related genes (Kosawang et al., 2014). As reported herein, enhancing library coverage using RNAseq instead of an EST library, and treating *C. rosea* with *F. graminearum-*spent culture media (containing live macroconidias) to mimic both environmental cues and *in situ* fungi-fungi interaction, provided a library that better represents transcriptomic changes associated with the mycoparasitism of *C. rosea*. While the composition of the *F. graminearum* secretome is unknown, and its' profiling goes beyond the scope of this study, we anticipate it will contain an array of polar metabolites, NRPs, polyketides as well as primary metabolites including amino acids, sugars, proteins and polysaccharides. A recent search of the literature highlighted one report describing some of the possible proteins that are likely found in an *F. graminearum* secretome (Brown et al., 2012). Furthermore, in the *C. rosea*—*F. graminearum* interaction, is not clear whether it is just secreted metabolites or a combination of secreted metabolites and a physical interaction (Xue et al., 2014), that modulates C. rosea metabolism. Thus it was desirable to introduce a “physical *F. graminearum* entity” in addition to the Fg secretome, for *C. rosea* to interact with. However, to keep the system as simple as possible, *F. graminearum* macroconidias, rather than a growing inoculum plug, were included with the secretome. To limit *Fusarium* macroconidia germination and contamination of our library with *Fusarium* RNA, we added the *Fusarium* spent media containing the live macroconidias after allowing *C. rosea* to establish itself in the liquid culture for 5 days. ACM941 can suppress *Fusarium* macroconidia germination by more than 99% when inoculated at equal concentration, let alone when it is given a growth advantage (Hue et al., 2009). Consistent with this, *C. rosea* has also been shown to completely kill *S. sclerotiorum* during co-cultivation in liquid media (Sun et al., 2015). As such isolated RNA was expected to be free of *Fusarium* RNA. Finally, that >92–96% of the reads were successfully mapped to IK726 genome using TopHat further supports this assumption (data not shown).

In agreement with a previous report (Kosawang et al., 2014), treatment of *C. rosea* with DON-alone, despite its role as a pathogenesis factor in *Fusarium*, had minimal influence on differential expression of core secondary metabolite-related genes in the RNASeq derived library, and failed to induce expression of any entire gene cluster. Also consistent with this, DON treatment did not up-regulate expression of an abcg5 homolog (Crosea_g22712), although a slight increase in expression of a CYP55A3 homolog (Crosea_g8458) was detected. In comparison, treatment with *Fusarium* spent media containing 10^3^ live macroconidias per ml was highly effective in inducing up-regulation of secondary metabolite-related genes in *C. rosea* AMC941, as well as the entire clusters they reside in. In addition, unigenes encoding transporters accounted for 6% of the *Fusarium* spent medium-treated library (Figure 4A), but were for all intents missing in the DON library. Clearly DON-alone is not sufficient to up-regulate these genes, suggesting additional regulation. Considering the resources required to synthesize most fungal secondary metabolite-related genes, ranging from 4.5 to 6.5 kb for ABC transporters, 6–8 kb for PKSs and 7–12 kb for NRPSs, and their precursors, one can appreciate why fungi do not readily express these genes.

Interestingly, the degree of modulation of expression levels of secondary metabolite-related genes did not directly correlate with the degree of growth inhibition. However, this was not entirely unexpected because the minimal liquid medium used (Czapek Dox), is likely not able to support the precursor demand of up-regulated genes. For example, two of the up-regulated putative gene clusters contain NRPS-like canonical enzymes, that require significant amounts of amino acids as precursors, which are not readily available in the minimal medium. It is also possible that some of the highly up-regulated genes, particularly PKSs, are involved in fungal development and/or protection as well. For example, two up-regulated PKSs in *C. rosea* IK726 upon interaction with *F. graminearum* were previously identified; however deletion of one led to pigmentation loss, while deletion of the other reduced the antagonistic activity of *C. rosea* IK726 against *F. graminearum* (Fatema, 2017). Secondary metabolism-related secondary metabolites have also been associated with fungal sclerotial development (Calvo and Cary, 2015).

*C. rosea* exposure to *Fusarium-*spent media containing live macroconidias resulted in the identification of three up-regulated putative secondary metabolite biosynthesis gene clusters. Two clusters have PKSs at their core, although AntiSMASH-fungal version (Medema et al., 2011) analysis of both sequences failed to predict any possible polyketide product (data not shown), implying their uniqueness. The first cluster contains a PKS that is surrounded by up-regulated genes putatively involved in the synthesis of acetyl-CoA from fatty acid, likely contributing to precursor biosynthesis to enable production of the PKSs. Interestingly, the gene cluster was intervened by Crosea_g17653—a cutinase homolog—that was not up-regulated in our RNAseq libraries (data not shown). As shown in β-lactam antibiotics biosynthesis in *A. chrysogenum*, intervention of gene clusters by non-member gene is not uncommon in fungi (Gutiérreze et al., 1999). The second cluster contains an up-regulated, highly-reducing, PKS homolog that is surrounded by unigenes putatively encoding an NRPS-like enzyme, an ABC transporter, sugar transferases and an alcohol dehydrogenase (Figure 5A). Based on these annotated functions, cluster 2 is proposed to be a putative TMC-151 family metabolite biosynthetic gene cluster. The TMC-151 family of metabolites are antifungal polyketides, exclusive to *C. rosea*, and notable for their high degree of C-methylation (up to nine methyl groups) and presence of sugar and hexitol or pentitol moieties (Okuda et al., 2000; Zhai et al., 2016). Feeding studies reported elsewhere have demonstrated that the C-methyl moieties of TMC-151 are derived from methionine, while the carbon chains are made up of ten acetate molecules (Kohno et al., 2000). Although rare, NRPS-like enzymes have been shown to function as C-methyl-transferases during polyketide synthesis, with the methyl groups derived from amino acids. For example, the NRPS module of the yersiniabactin biosynthetic cluster core hybrid NRPS/PKS from *Yersinia pestis* catalyzes methylation of a thiozolinyl-S-peptidyl carrier protein intermediate (Miller et al., 2001). The fact that the methyl groups of the TMC-151 family metabolites are derived from methionine and a putative NRPS-like transcript is up-regulated in the gene cluster provided added confidence in assigning the putative function of this cluster. Consistent with this, sequence analysis of the NRPS-like homolog using the SBSPKS *v*2 online tool (Khater et al., 2017) showed that it is composed of three domains: a formyl transferase, as well as domains involved in adenylation and thiolation (data not shown). Also, although the PKS and NRPS genes were putatively encoded as separate transcripts in all assembly results and genomic-based analysis, it is entirely possible that they are encoded as a hybrid enzyme as well.

Recently an isoquinoline alkaloid was characterized from *A. fumigatus* and called fumisoquins (Baccile et al., 2016). The gene cluster responsible for its biosynthesis is composed of genes encoding an NRPS-like enzyme, an ATP-grasp enzyme, an MDR-type ABC transporter, and a phenol 2-monooxygenase. This is highly reminiscent of the third up-regulated gene cluster identified in this study, with the genes sharing a high degree of sequence similarity with each other. Thus, cluster 3 is proposed to be a *C. rosea* fumisoquin-like biosynthetic gene cluster. Isoquinoline alkaloids, synthesized by both plants and fungi, have shown promising antimicrobial properties. For example, fusarmine is a polyketide isoquinoline alkaloid from the endophytic fungus *Fusarium* sp. LN12 that is under investigation for its antimicrobial property (Yang et al., 2012).

In conclusion, a transcriptomic library of *C. rosea* ACM941 has been developed and gene clusters putatively involved in the synthesis of antifungal metabolites in liquid culture were identified. The up-regulation of these clusters, that would otherwise not have been detected using only purified *Fusarium* mycotoxin treatments, was achieved with the addition of *Fusarium-*spent media and a low concentration of live macroconidias. While functional validation of the identified clusters is currently underway, the library also contains a range of other genes whose functional elucidation could shed more light on the lifestyle of *C. rosea*. Ultimately, this library will serve as a platform to develop a more complete understanding of the mode of action enabling *C. rosea's* bio-control activity, information that can eventually be used to develop more potent strains or metabolites that can serve as novel lead anti-fungal compounds.

## Author contributions

ZD conceived some of the ideas, planned and carried out all the experiments, analyzed and interpreted the data, and wrote the first draft of the paper. SF and YT helped plan experiments and contributed to data processing and analysis. ML conceived of some of the ideas, oversaw the planning of experiments, interpreted the data and wrote the final version of the paper.

### Conflict of interest statement

The authors declare that the research was conducted in the absence of any commercial or financial relationships that could be construed as a potential conflict of interest.

## Abbreviations

BCA(s): biological control agent(s)
DON: deoxynivalenol
FHB: *Fusarium* head blight
MeOH: methanol
NRPS(s): non-ribosomal peptide synthase(s)
PKS(s): polyketide synthase(s).
