# Supplementary material for: Profiling of the Transcriptomic Responses of Clonostachys rosea Upon Treatment With Fusarium graminearum Secretome
Source: Front Microbiol. 2018 Jun 7;9:1061. doi: 10.3389/fmicb.2018.01061 (PMC5999785; doi:10.3389/fmicb.2018.01061)
Supplement: Supplementary file 1 [file Presentation_1.PDF]

Profiling of the transcriptomic responses of *Clonostachys rosea* upon treatment with *Fusarium graminearum* secretome

Zerihun A. Demissie[1], Simon Foote[2], Yifang Tan[3] and Michele C. Loewen[1,4,*]

[1] Aquatic and Crop Resource Development, National Research Council Canada, 100 Sussex Dr., Ottawa, ON, Canada
[2] Human Health Therapeutics, National Research Council of Canada, 100 Sussex Dr., Ottawa, ON, Canada
[3] Aquatic and Crop Resource Development, National Research Council Canada, 110 Gymnasium place, Saskatoon, SK, Canada
[4] Department of Biomedical and Molecular Sciences, Queens University, Kingston, ON, Canada

*Corresponding Author: Michele Loewen, michele.loewen@nrc.ca

A

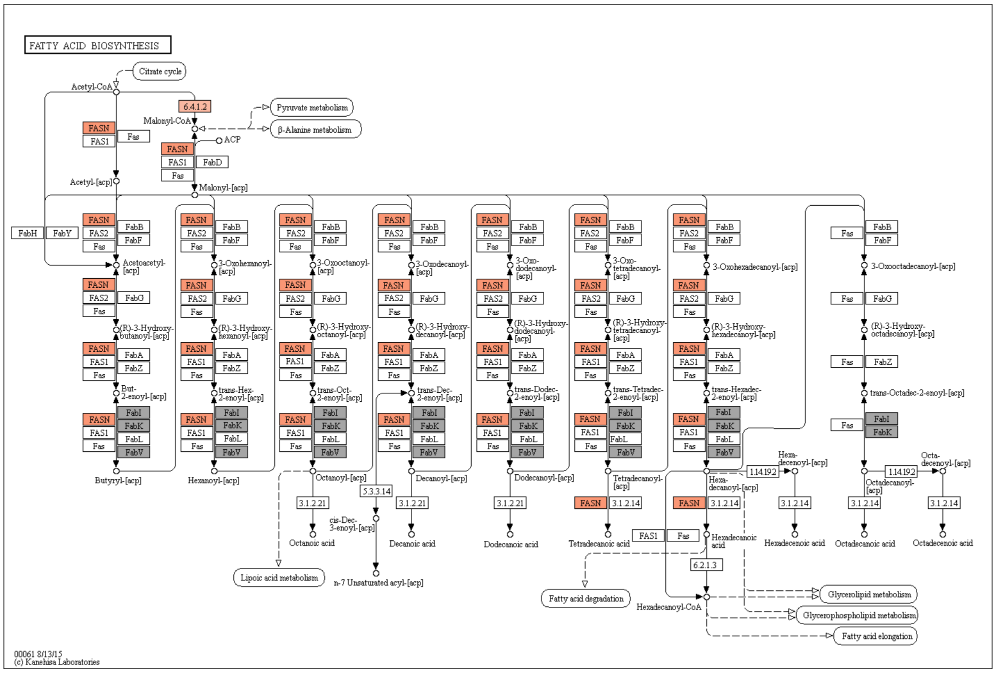

B

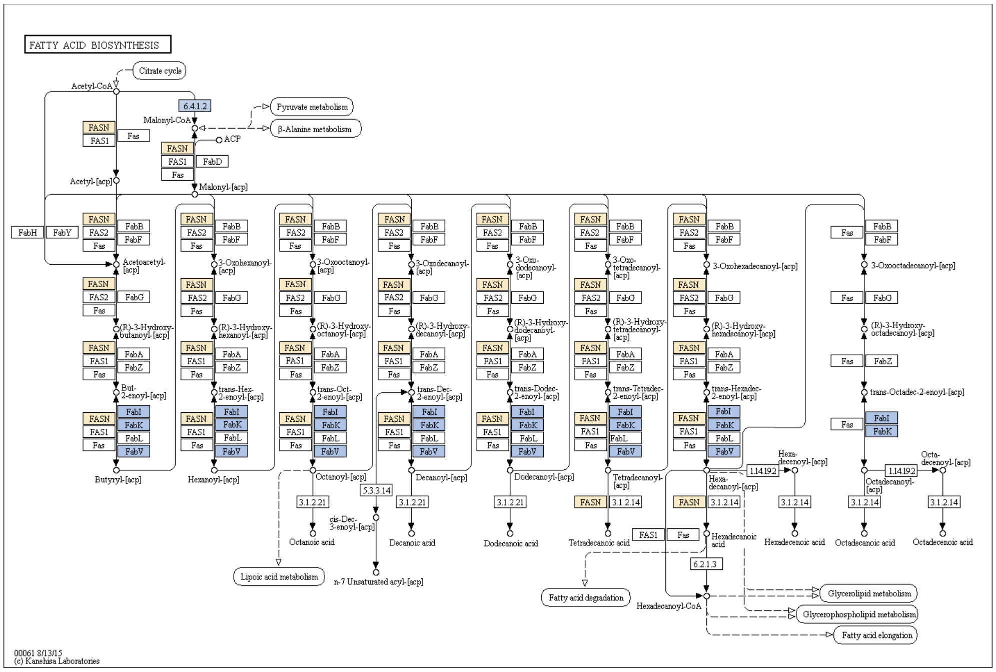

Supplementary Figure 1. KEGG mapping of up-regulated library on fatty acid biosynthesis pathway. A) *Fusarium*-spent media containing live spore treated *C. rosea* relative to MeOH and B) DON treated *C. rosea* relative to MeOH.

Supplementary Table 1: List of top hit Blast species.

No	**Species**	**%**	**Life style**	**Common feature**
1	*Acremonium chrysogenum* ATCC 11550	13.2	Saprophyte	Secondary metabolite biosynthesis
2	*Neonectria ditissima*	5.9	Nectrotrophic	
3	*Nectria haematococca* mpVI 77-13-4	5.7	Pathogen	Secondary metabolite biosynthesis and antibiotic tolerance
4	*Purpureocillium lilacinum*	2.4	Endophyte	BCA/ Secondary metabolite biosynthesis
5	*Tolypocladium ophioglossoides* CBS 100239	2.3	Parasitic	BCA/medicinal value
6	*Schizosaccharomyces pombe* 972h-	1.9	-	
7	*Pochonia chlamydosporia* 170	1.6	Nematophagus	BCA
8	*Stachybotrys chlorohalonata* IBT 40285	1.6	-	mycotoxin producers
9	*Stachybotrys chartarum* IBT 7711	1.4		mycotoxin producers
10	*Stachybotrys chartarum* IBT 40293	1.4		mycotoxin producers
11	*Stachybotrys chartarum* IBT 40288	1.4		mycotoxin producers
12	*Pestalotiopsis fici* W106-1	1.3	Endophytic	Secondary metabolite biosynthesis
13	*Fusarium avenaceum*	1.1	Pathogen	mycotoxin producers
14	*Saccharomyces cerevisiae* S288c	1.1		
15	*Trichoderma virens* Gv29-8	1.0	Mycoparasitism	BCA
16	*Eutypa lata* UCREL1	1.0	Pathogen	
17	*Hirsutella minnesotensis* 3608	0.9	Nematophagus	BCA
18	*Trichoderma harzianum*	0.8	Mycoparasitism	BCA/ Secondary metabolite biosynthesis
19	*Colletotrichum gloeosporioides* Cg-14	0.8	Pathogen	
20	*Colletotrichum gloeosporioides* Nara gc5	0.8	Pathogen	

Supplementary Table 2: Differential expression of top 20 hit members, sorted based on CBS12511 RNAseq analysis in all expression libraries.

**CBS125111 extracted transcript library ID**	**A**	**B**	**IK726-trasncript library ID**	**A**	**B**	**ACM941 Trinity assembled library ID**	**A**	**B**	**ACM941 CLC-assembled library ID**	**A**	**B**	**Homology description**
XLOC_018259	NA	2591	BN869_T00007381_1	63.5	2933	Crosea_g5068	62.6	2840	Contig_1989	49.6	2387	polyketide synthase
XLOC_002184	NA	2202	NA	NA	NA	Crosea_g12508	2	187	Contig_1528	11.8	1942	polyketide synthase
XLOC_008277	NA	269.755	BN869_T00013027_1	2.4	4.1	Crosea_g11038	2.2	3.277	Contig_1617	2	3.74	Thiamine thizole synthase
XLOC_017405	81.8	184.4	BN869_T00004217_1	10.9	30.4	Crosea_g11869	1.6	1.1	Contig_1856	3.5	8.7	sulfate permease
XLOC_009873	NA	171.914	BN869_T00011141_1	0.6	2.45	Crosea_g636	3.5	1.31				Related to carboxypeptidase A
XLOC_003580	7	148.698	BN869_T00001019_1	3.8	44.5	Crosea_g7327	1.4	8	Contig_11114	2.7	22.8	Ammonium transporter MEAA
XLOC_009494	29.4	130.812	BN869_T00009374_1	13.1	30.2	Crosea_g6554	11.3	22	Contig_8188	11.3	23.3	4-coumarate:coenzyme A ligase
XLOC_002972	NA	125.866	BN869_T00005209_1	3.1	15	Crosea_g9244	1	2.4	Contig_1789	1.84	-1.12	Urea active transporter
XLOC_013801	NA	111.593	BN869_T00007922_1	5.8	33.6	Crosea_g6661	6	34	Contig_109	5.6	33.6	Insect inhibitor with A fungal trypsin
XLOC_006656	NA	68.253	NA			Crosea_g17346	1.8	248	Contig_5389	1.2	213	Ethanolamine utilization
XLOC_008278	NA	56.267	BN869_T00013028_1	1.5	3.4				Contig_1617	2.1	3.7	Mitochondrial triosephosphate isomerase
XLOC_010218	NA	47.979	BN869_T00007803_1	4.4	2.7	Crosea_g13197	2	1.3	Contig_23047	4.8	3.6	IMP-specific 5-nucleotidase 1
XLOC_016265	NA	47.977	BN869_T00005706_1	2.4	19	Crosea_g4245	2.4	18.5	Contig_6695	2.1	16.8	Aquoporine-1
XLOC_009400	NA	45.214	BN869_T00010116_1	NA	108	Crosea_g6309	0.4	71	Contig_539	-2.7	69.2	Agmatinase 1
XLOC_004207	NA	45.042	NA			Crosea_g7677	1.4	3.5	Contig_22544	7.2	14.3	Sorbitol dehydrogenase
XLOC_004791	NA	42.403	NA			Crosea_g12541	0.8	0.6	Contig_12606	-1.5	-1.9	NUDIX domain containing
XLOC_002833	NA	42.39	BN869_T00002751_1	2.1	1.6	Crosea_g10817	2.1	1.6	Contig_10653	2	1.8	Beta-glucosidase b
XLOC_018801	NA	39.7	BN869_T00008748_1	2	4.9	Crosea_g7462	2	5	Contig_3181	1.8	4.7	multidrug resistance transporter
XLOC_007732	NA	39.688	BN869_T00004377_1	1	1	Crosea_g10493	1.1	0.5	Contig_4448	1	1	Sterol glucosyltransferase
XLOC_016282	1.8	37.919	BN869_T00005687_1	1.6	26	Crosea_g7300	1.2	10.6	Contig_1988	1.4	15.2	Ammonium transporter 1
XLOC_005130	NA	37.093	BN869_T00008079_1	5.71	33.4	Crosea_g11018	6	34	Contig_4015	5.9	35	Serine endopeptidase

Abbreviations – **A**: expression in response to DON treatment relative to methanol and **B**: expression in response to *Fusarium*-spent media containing live spores treatment relative to methanol. NA: not available.
